# Deciphering key genes involved in cisplatin resistance in kidney renal clear cell carcinoma through a combined *in silico* and *in vitro* approach

**DOI:** 10.32604/or.2023.030760

**Published:** 2023-09-15

**Authors:** MUNEEBA MALIK, MAMOONA MAQBOOL, TOOBA NISAR, TAZEEM AKHTER, JAVED AHMED UJAN, ALANOOD S. ALGARNI, FAKHRIA A. AL JOUFI, SULTAN SHAFI K. ALANAZI, MOHAMMAD HADI ALMOTARED, MOUNIR M. SALEM BEKHIT, MUHAMMAD JAMIL

**Affiliations:** 1 Services Hospital, Lahore, Pakistan; 2 Sabzazar Medical Center, Lahore, Pakistan; 3 BHU Pourmiana, Attock, Pakistan; 4 Public Health Department, University of Health Sciences, Lahore, Pakistan; 5 Department of Zoology, Shah Abdul Latif University, Khairpur, Pakistan; 6 Department of Animal Sciences, University of Florida, Gainesville, USA; 7 Pharmacology and Toxicology Department College of Pharmacy, Umm Al-Qura University, Makkah, Saudi Arabia; 8 Department of Pharmacology, College of Pharmacy, Jouf University, Aljouf, Saudi Arabia; 9 Regional Laboratory and Blood Bank (Total Laboratory Automation), Riyadh, Saudi Arabia; 10 Health Affairs in Najran, New Najran General Hospital, Najran, Saudi Arabia; 11 Department of Pharmaceutics, College of Pharmacy, King Saud University, Riyadh, Saudi Arabia; 12 PARC Arid Zone Research Center, Dera Ismail Khan, Pakistan

**Keywords:** KIRC, Cisplatin resistance, Chemotherapy, Overall survival

## Abstract

The low survival rate of Kidney renal clear cell carcinoma (KIRC) patients is largely attributed to cisplatin resistance. Rather than focusing solely on individual proteins, exploring protein-protein interactions could offer greater insight into drug resistance. To this end, a series of *in silico* and *in vitro* experiments were conducted to identify hub genes in the intricate network of cisplatin resistance-related genes in KIRC chemotherapy. The genes involved in cisplatin resistance across KIRC were retrieved from the National Center for Biotechnology Information (NCBI) database using search terms as “Kidney renal clear cell carcinoma” and “Cisplatin resistance”. The genes retrieved were analyzed for hub gene identification using the STRING database and Cytoscape tool. Expression and promoter methylation profiling of the hub genes was done using UALCAN, GEPIA, OncoDB, and HPA databases. Mutational, survival, functional enrichment, immune cell infiltration, and drug prediction analyses of the hub genes were performed using the cBioPortal, GEPIA, GSEA, TIMER, and DrugBank databases. Lastly, expression and methylation levels of the hub genes were validated on two cisplatin-resistant RCC cell lines (786-O and A-498) and a normal renal tubular epithelial cell line (HK-2) using two high throughput techniques, including targeted bisulfite sequencing (bisulfite-seq) and RT-qPCR. A total of 124 genes were identified as being associated with cisplatin resistance in KIRC. Out of these genes, MCL1, IGF1R, CCND1, and PTEN were identified as hub genes and were found to have significant (*p* < 0.05) variations in their mRNA and protein expressions and effects on the overall survival (OS) of the KIRC patients. Moreover, an aberrant promoter methylation pattern was found to be associated with the dysregulation of the hub genes. In addition to this, hub genes were also linked with different cisplatin resistance-causing pathways. Thus, hub genes can be targeted with Alvocidib, Estradiol, Tretinoin, Capsaicin, Dronabinol, Metribolone, Calcitriol, Acetaminophen, Acitretin, Cyclosporine, Azacitidine, Genistein, and Resveratrol drugs. As the pathogenesis of KIRC is complex, targeting hub genes and associated pathways involved in cisplatin resistance could bring a milestone change in the drug discovery and management of drug resistance, which might uplift overall survival among KIRC patients.

## Introduction

Kidney renal clear cell carcinoma (KIRC) is a type of kidney cancer that originates from the cells lining the small tubules in the kidney that filter waste from the blood [1]. KIRC is the most common subtype of renal cell carcinoma, accounting for approximately 75% of all cases [2]. The incidence of KIRC has been increasing over the past few decades [3], and it is estimated that there will be approximately 76,080 new cases of kidney cancer in the United States in 2022, with KIRC accounting for the majority of cases [4].

The prognosis for KIRC varies based on factors such as Tumour, Node, Metastasis (TNM) staging, age, and gender, with the patient’s five-year survival rate heavily influenced by the tumor’s pathological grade [5,6]. KIRC patients with an early-stage tumor (grade I or II) tend to have better treatment outcomes and a longer survival period post-surgery, with a survival rate of 80%–90% [7]. Conversely, those diagnosed in the advanced stages of the disease (grade III or IV) have a poorer prognosis, with a survival rate of around 20% due to the high malignancy and probability of metastasis or recurrence [8]. Risk factors for KIRC include smoking, obesity, high blood pressure, and a family history of kidney cancer. KIRC is often asymptomatic in its early stages, and symptoms may not appear until the disease has advanced. Common symptoms of KIRC include blood in the urine, back pain, weight loss, and fatigue [9,10].

The management of KIRC involves a variety of approaches, including surgery, radiotherapy, chemotherapy, and immunotherapy [11–13]. Chemotherapy is considered a foundational aspect of cancer treatment and is frequently used alone or in conjunction with radiation therapy for locally advanced tumors [14]. Cisplatin is a commonly used chemotherapeutic drug for managing KIRC that operates by creating DNA adducts and disrupting cell cycles, leading to cell death [15,16]. For locally advanced tumors (stages III and IV), cisplatin is usually given during concurrent chemoradiation therapy (CRT), either alone or following surgery, or as induction therapy followed by CRT [17]. In cisplatin-based CRT, the drug is administered at a dosage of 100 mg/m^2^ IV on days 1, 22, and 43, or at a weekly dose of 30-40 mg/m^2^ IV for 6 to 7 weeks [18]. Despite all this treatment modality, only around 50% of locally advanced KIRC patients respond to therapy [19].

Earlier, several studies reported the development of cisplatin resistance in KIRC patients. In a study by Taniguchi et al. they found that cisplatin resistance was associated with the overexpression of the MDR1 gene in KIRC cells [20]. Similarly, Liu et al. reported that high level of miR-193a-3p expression was associated with cisplatin resistance in KIRC patients [21]. Other studies, such as the one by Sui et al. identified the role of autophagy in cisplatin resistance development in KIRC cells [22]. These findings suggest that cisplatin resistance in KIRC could be due to multiple factors, and the underlying mechanisms need to be further investigated to overcome this challenge.

Moreover, cancer patients who are resistant to cisplatin treatment typically have a bleak prognosis [23]. Proteins are vital macromolecules involved in various cellular operations, and understanding their expression and interactions can provide insight into the complicated molecular pathways involved in drug resistance [24]. Protein-protein interaction (PPI) is an indirect phenomenon that contributes to various cellular functions [25]. While transient PPI controls signaling pathways, permanent PPI forms a protein complex. Approximately 80% of protein functions occur through PPI [26], making the assessment of protein complexes or networks of proteins a more effective approach than targeting a single protein in defeating drug resistance. Furthermore, PPI interactions can help predict the role of previously unexplained proteins.

Hence, this study was initiated for identifying biomarker hub genes through *in silico* and *in vitro* methodologies, that are associated with cisplatin resistance in KIRC. *In silico* and *in vitro* combined approach for identifying cisplatin resistance-associated hub genes is the integration of computational and experimental methods. The *in silico* approach involves the utilization of bioinformatics tools and algorithms to analyze large-scale genomic, transcriptomic, and proteomic data sets. On the other hand, the *in vitro* approach involves the application of experimental methods to validate the identified hub genes, including their expression and methylation levels. The integration approach of *in silico* and *in vitro* methodologies can help improve our understanding of cisplatin resistance development in KIRC, leading to the development of more effective therapies.

## Materials and Methods

### Extraction of cisplatin resistance-associated genes in KIRC

**Figure 1:**
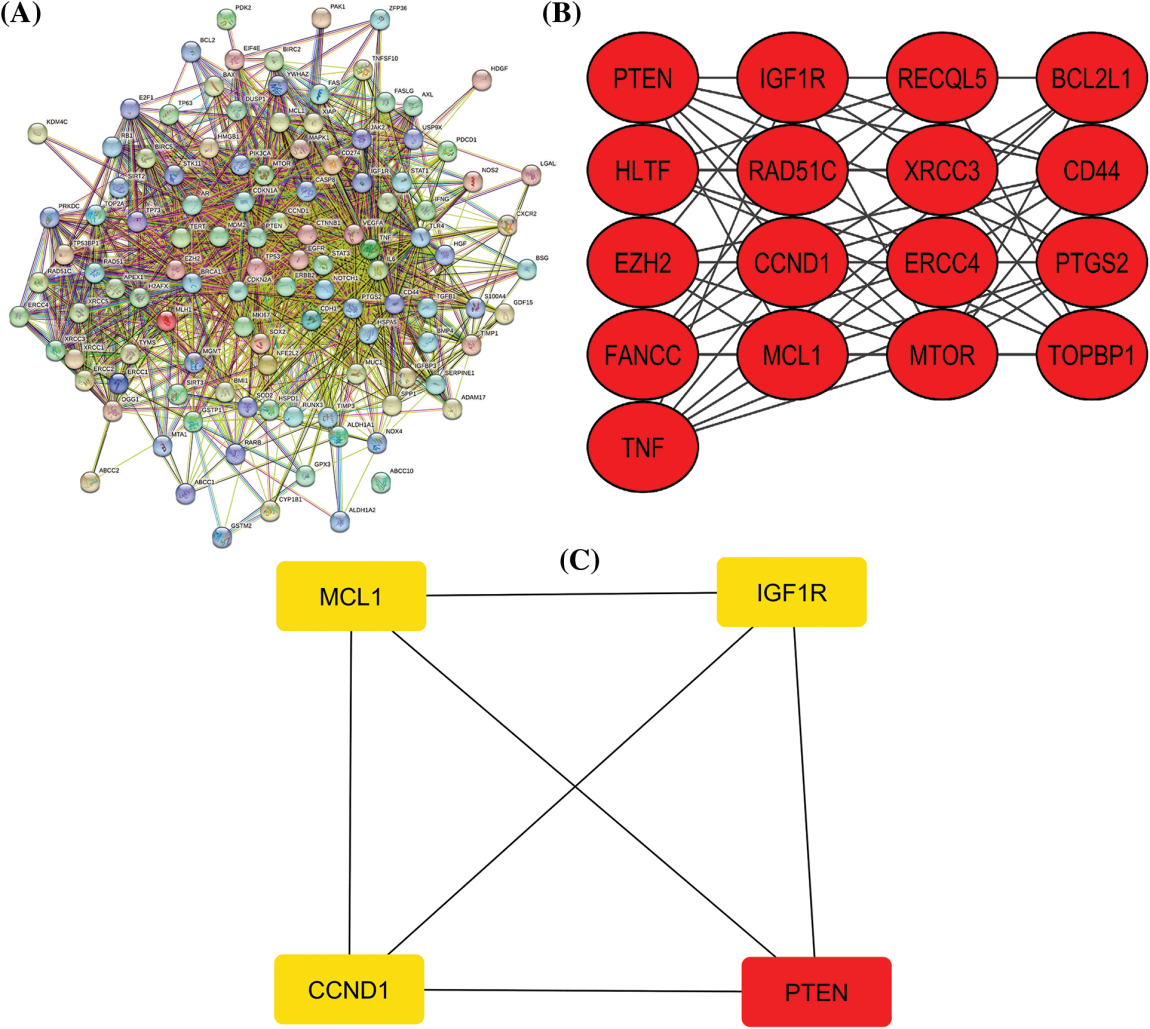
A PPI network of the cisplatin resistance-related genes, a significant module in the constructed PPI network, and a PPI network of the identified hub genes. (A) A PPI network of the cisplatin resistance-related genes, (B) a PPI network of the most significant module, and (C) a PPI network of identified four hub genes.

The National Center for Biotechnology Information (NCBI, https://www.ncbi.nlm.nih.gov/) database [27] was searched to identify cisplatin resistance-associated genes in KIRC patients. The search term included “Kidney renal clear cell carcinoma” AND “Cisplatin resistance”. At the end of the search process, a total of 191 items related to *Homo sapiens* have appeared. Out of the appeared items, a total of 124 cisplatin resistance genes were used in the present work (Fig. 1A). Detail of the search further includes: (Kidney renal clear cell carcinoma [All Fields], (Cisplatin [All Fields], Resistance [All Fields]), “*Homo sapiens*” [porgn].

### PPI and cisplatin resistance-related hub gene identification

STRING (https://string-db.org/) [28] is a database and web resource that provides a comprehensive collection of known and predicted protein-protein interactions. Using this data, researchers can construct protein association networks that help identify potential functional relationships between proteins. These networks can assist in understanding biological mechanisms and disease processes, as well as identifying potential drug targets. STRING also provides tools for network visualization and analysis, allowing researchers to explore the relationships between proteins more deeply [28]. Overall, STRING is a valuable resource for researchers studying protein function and interaction networks. In this work, we used the STRING web resource for constructing the protein-protein interaction (PPI) network of the cisplatin resistance proteins with the default settings. Cystoscope [29] is an open-source platform designed for the analysis of complex networks. With its user-friendly interface and powerful algorithms, Cystoscope makes it easy for researchers and data analysts to explore large-scale networks and uncover hidden patterns and relationships [29]. Users can also perform network analysis tasks such as community detection, centrality analysis, and network comparison. Overall, Cystoscope is a valuable tool for anyone working with complex networks, and its open-source nature ensures that it will continue to evolve and improve over time. MCODE plugin [30] of the Cytoscape platform is an algorithm, which is used to identify densely connected regions or modules in a PPI network. The MCODE algorithm works by first identifying dense regions or clusters by calculating the density of a node’s neighborhood. It then scores the clusters based on their size and density, and the top-scoring clusters are returned as the output. The algorithm considers various factors, including the number of nodes and edges, whether there is a high degree of connectivity within a cluster, and whether a cluster is densely connected compared to the rest of the network. MCODE was used in this tool to identify significant module in the constructed PPI network of the cisplatin resistance-associated genes. Cytohubba is another plugin of the Cytoscape software that is used to identify hub genes in a PPI network [30]. Cytohubba utilizes various topological measures, including the degree of centrality to understand the significance of a gene node in the network. These measures help identify the most connected genes in the network, which are considered hub genes. This source was used in the present study to identify hub genes from the constructed PPI network of the cisplatin resistance-associated genes based on the degree method.

### mRNA and protein expression profiling of hub genes

UALCAN (https://ualcan.path.uab.edu/analysis-prot.html) [31] is an online database that provides gene expression analysis of cancer data from The Cancer Genome Atlas (TCGA) project. The database is designed to be user-friendly and accessible for researchers and clinicians who want to study the expression of genes and proteins in different cancer types [31]. UALCAN allows users to search for a specific gene or protein of interest and visualize its expression levels across different cancer types, subtypes, and clinical characteristics [31]. UALCAN is a powerful tool for cancer research and personalized medicine, and its open-access nature makes it a valuable resource for the scientific community. UALCAN was used in this work for mRNA and protein expression profiling of the cisplatin resistance-associated hub genes across KIRC samples relative to controls.

### mRNA expression validation and survival analysis of hub genes using additional TCGA detests

GEPIA (http://gepia.cancer-pku.cn/) [32] and OncoDB (https://oncodb.org/index.html) [33] are widely used web-based tools that allow researchers to perform gene expression analysis on different types of cancer as well as normal tissues. These tools provide an interactive interface for users to explore and visualize gene expression patterns, differential expression analysis, and survival analysis. These databases include RNA sequencing data from The Cancer Genome Atlas, providing comprehensive resource for cancer research. The tool also integrates functional enrichment analysis, which allows users to identify enriched gene ontology terms and biological pathways associated with differentially expressed genes. In conclusion, both GEPIA and OncoDB tools have become essential tools for cancer research, providing researchers with a comprehensive platforms to analyze gene expression patterns, identify potential therapeutic targets, and develop personalized cancer treatments. In this work, the GEPIA and OncoDB databases were used for the expression validation analysis of the hub genes across KIRC samples relative to controls. Moreover, GEPIA database was further utilized for survival analysis as well.

### Subcellular localization and protein expression validation of hub genes

The Human Protein Atlas (HPA, https://www.proteinatlas.org/) database is an extensive and accessible repository of information on the human proteome [34]. It comprises a comprehensive collection of information on tissue and cell-specific protein expression patterns in humans, providing an invaluable resource for researchers and scientists around the world [34]. The database leverages a wide range of cutting-edge technologies, including high-throughput proteome analysis, antibody-based profiling, and molecular-imaging techniques. Additionally, the HPA offers interactive tools and resources for data visualization and analysis, enabling users to explore and interpret the data in various ways. In this work, the HPA database was used to identify the subcellular localization of the proteins encoded by the hub genes in KIRC cells as well as to validate hub gene expression at the protein level across KIRC samples relative to controls based on the immunohistochemical images.

### Methylation analysis of hub genes

UALCAN [31] and OncoDB [33] databases were used in the present work for the methylation analysis of the hub genes across KIRC samples relative to controls.

### Mutational analysis, mutation based survival analysis, and co-express gene analysis of hub genes

cBioPortal (https://www.cbioportal.org/) is a user-friendly, open-access platform that enables researchers and clinicians to perform complex genomic analysis on cancer data [35]. It offers visualization and analysis tools for studying complex cancer genomics data, allowing users to explore and analyze large-scale clinical datasets with ease. The database includes genomic data from over 270 cancer studies, including more than 30,000 patients across 20 different types of cancer [35]. The platform incorporates multiple tools, such as data visualization, differential expression analysis, and gene set enrichment analysis, that enable researchers to query the database in various ways. The ability to stratify data by patient characteristics and cancer subtype greatly enhances the potential for personalized cancer treatment. cBioPortal provides an invaluable resource of genomic data for researchers and clinicians, helping revolutionize cancer diagnosis and treatment. In this work, this tool was used for the mutational analysis, mutation-based survival analysis, and co-express gene analysis of hub genes in TCGA KIRC samples.

### Functional enrichment analysis

The Gene Ontology (GO) analysis provides functional annotation of the gene(s) of interest. While KEGG (Kyoto Encyclopedia of Genes and Genomes) provides an interpretation of the user-defined genes in biological pathways [36]. The GO and KEGG analyses of the hub and hub gene-enriched genes were performed using the GSEA program [37].

### Immune cell infiltration analysis

The TIMER database (http://timer.cistrome.org/) is a comprehensive tool that allows for the estimation of immune cells in a variety of tumor types [38]. The database was developed by researchers at the University of Texas MD Anderson Cancer Center and provides a wealth of information on the immune status of tumors, including the abundance of various immune cells, such as T cells, B cells, natural killer cells, and dendritic cells [38]. This information can be used to better understand the immune response to tumors and develop more effective cancer treatments. In this research, levels of immune cell infiltration in KIRC were plotted against hub gene expression.

### miRNA network analysis

The ENCORI platform (https://rna.sysu.edu.cn/encori/) was developed for analyzing the miRNA-mRNA, RNA-RNA, and lncRNA-miRNA networks across various types of cancer [39]. In this investigation, the ENCORI database was used to create the miRNA network of the identified hub genes [39].

### Hub genes’ drug prediction analysis

We used the DrugBank database (https://go.drugbank.com/) to uncover a variety of drugs associated with the identified hub genes because we believe that the identified hub genes could be promising therapeutic targets. This database compiles information on chemotherapeutic drugs that target hub genes from a variety of reliable sources [40].

### In vitro validation of cisplatin resistance-inducing genes via targeted bisulfide-seq and RT-qPCR analyses

Cell lines: Human RCC cell lines (786-O and A-498) and a normal renal tubular epithelial cell line (HK-2) were purchased from the American Type Culture Collection (ATCC, USA) and cultivated in accordance with the manufacturer’s instructions.

Construction of cisplatin resistance cell strains: Cisplatin resistance of cells was induced using a previously established pulse therapy technique [41] combined with continuous stepwise exposure to the drug. Finally, the cisplatin resistance cells were acquired at a cisplatin concentration of 0.5 μg/mL.

Total RNA and DNA extraction: Total RNA extraction from both KIRC and normal cell lines was done by isopycnic centrifugation as described previously [42]. The extracted RNA was then processed for the DNA digestion step of incubation with RNase-free DNase I (Roche, Germany) at 37°C for 15 min. The DNA extraction was done following the organic method [43]. The quality of the extracted RNA and DNA was checked by a 2100 Bioanalyzer (Agilent Technologies, Germany).

Targeted bisulfite-seq and RT-qPCR analyses: DNA samples were sent to Beijing Genomics Institute (BGI) company for targeted bisulfite-seq analysis. Following targeted bisulfite-seq analyses, methylation values were normalized as beta values. The obtained beta values against hub genes in RCC and a normal control cell line were compared to identify differences in the methylation levels.

RT-qPCR validation analysis: The specific protocols are as follows: First, the PrimeScript™ RT reagent kit (Takara, Japan) was used for reverse transcription of the extracted RNA from cisplatin-resistant RCC cell lines (786-O and A-498) and a normal control cell line (HK-2) into complementary DNA. Then, the RT-qPCR was carried out on an ABI ViiA 7 Real Time PCR System (Thermo Fisher, USA) with a SuperReal SYBR Green Premix Plus (Tiangen Biotech, China) as a fluorescent dye. GAPDH was chosen as the internal reference in the present study. All the experiments were done in triplicate independently. All the primers for each hub gene are shown as following:

GAPDHF 5-ACCCACTCCTCCACCTTTGAC-3′,

GAPDHR 5′-CTGTTGCTGTAGCCAAATTCG-3′ [44].

MCL1F 5′-GGACATCAAAAACGAAGACG-3′,

MCL1R 5′- GCAGCTTTCTTGGTTTATGG-3′ [45].

IGF1RF 5′-GGCACAATTACTGCTCCAAAGAC-3′,

IGF1RR 5′-CAAGGCCCTTTCTCCCCAC-3′ [46].

CCND1F 5′-CGATGCTGGAGGTCTGCGA-3′,

CCND1R 5′-AGAGGCCACGAACATGCAAG-3′ [47].

PTENF 5′-TGGCATACACCAAATATAAGAGC-3′,

PTENR 5′-TCCCTTATCAGATACATGACTTTCA-3′ [48].

### Statistics details

For enrichment analysis, we used Fisher’s Exact test for computing statistical difference [49]. Correlational analyses were carried out using the Pearson method. The 2^−ΔΔCt^ method was employed to evaluate the relative expression of each hub gene through RT-qPCR [50]. Log-rank test was used to compare survival curves between normal control and KIRC patients. Cox proportional hazards model was utilized to study the relationship between survival time and the predictive variable [51]. For comparisons, a student *t*-test was adopted in the current study. All the analyses were carried out in R version 3.6.3 software.

## Results

### Extraction of cisplatin resistance-associated genes, PPI network, module analysis, and hub genes recognition

After a brief literature search, a total of 124 genes were found associated with cisplatin resistance across KIRC in *Homo sapiens*. The obtained 124 genes were evaluated for constructing the PPI network through the STRING database. As highlighted in Fig. 1A, the constructed PPI consisted of 124 nodes, where nodes and edges represent proteins and protein–protein associations, respectively. Further, the constructed PPI was analyzed using the MCODE plugin application of the Cytoscape software to identify a significant module consisting of 17 nodes (Fig. 1B). Then, the identified significant module was subjected to Cytohubba analysis for the identification of hub genes based on the degree method. Cytohubba analysis revealed MCL1 (myeloid cell leukemia-1), Insulin-like growth factor 2 receptor (IGF1R), CCND1 (Cyclin D1), and Phosphatase and TENsin homolog deleted on chromosome 10 (PTEN) genes were the hub genes having the highest degree scores (Fig. 1C).

### Expression and correlation analysis of MCL1, IGF1R, CCND1, and PTEN with different clinical variables using the TCGA dataset

**Figure 2:**
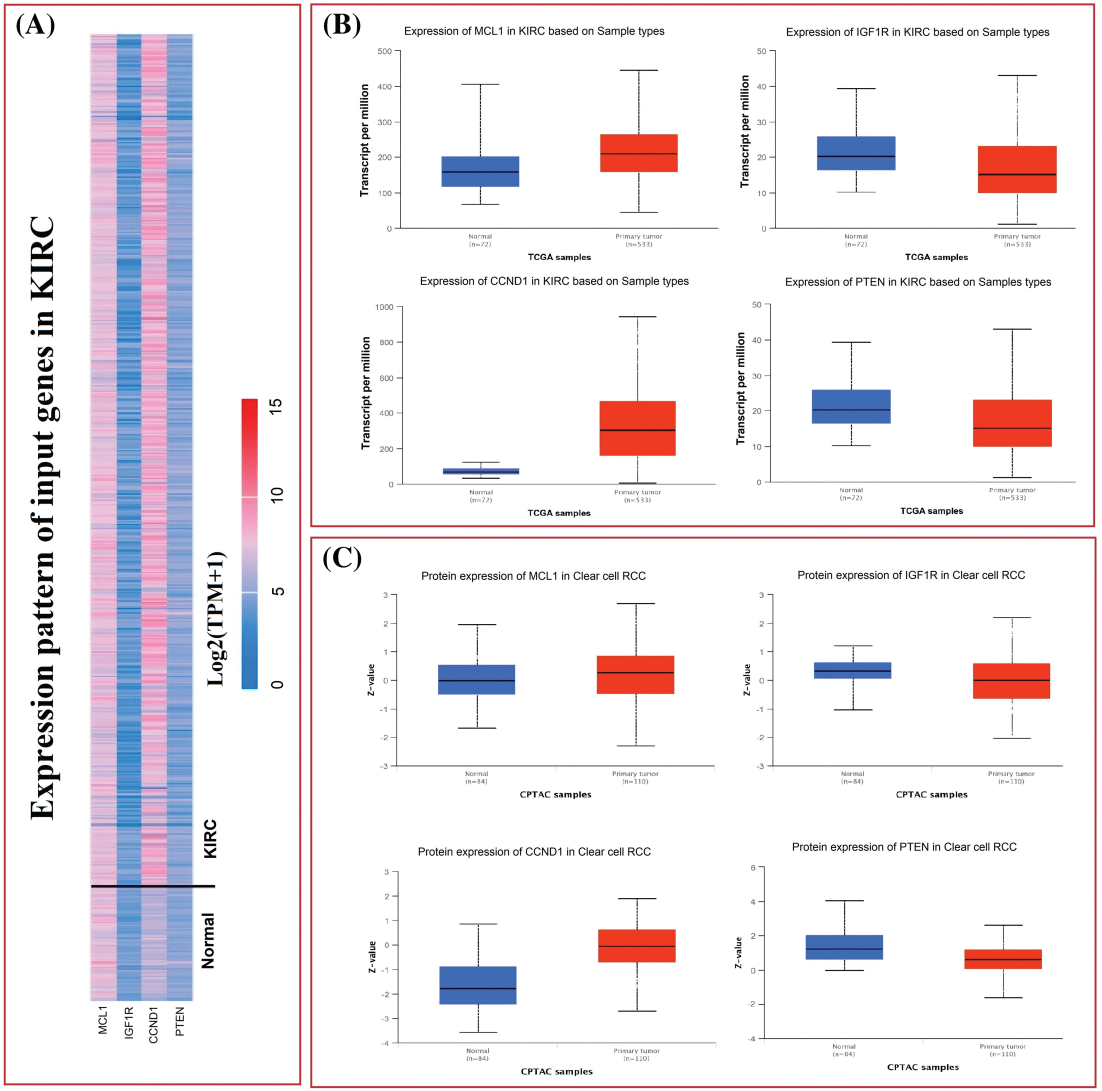
mRNA and protein expression profiling of MCL1, IGF1R, CCND1, and PTEN via UALCAN. (A) A heatmap of MCL1, IGF1R, CCND1, and PTEN hub genes in KIRC sample group and normal control group, (B) box plot presentation of MCL1, IGF1R, CCND1, and PTEN hub genes mRNA expression in KIRC sample group and normal control group, and (C) box plot presentation of MCL1, IGF1R, CCND1, and PTEN hub genes protein expression in KIRC sample group and normal control group.

The TCGA dataset was used in conjunction with the UALCAN tool to analyze the expressions of MCL1, IGF1R, CCND1, and PTEN. The results showed a significant up-regulation of MCL1 and CCND1, while IGF1R and PTEN showed a significant down-regulation at both mRNA and protein levels in KIRC samples compared to controls (Figs. 2A–2C). This difference was statistically significant (*p* < 0.05).

**Figure 3:**
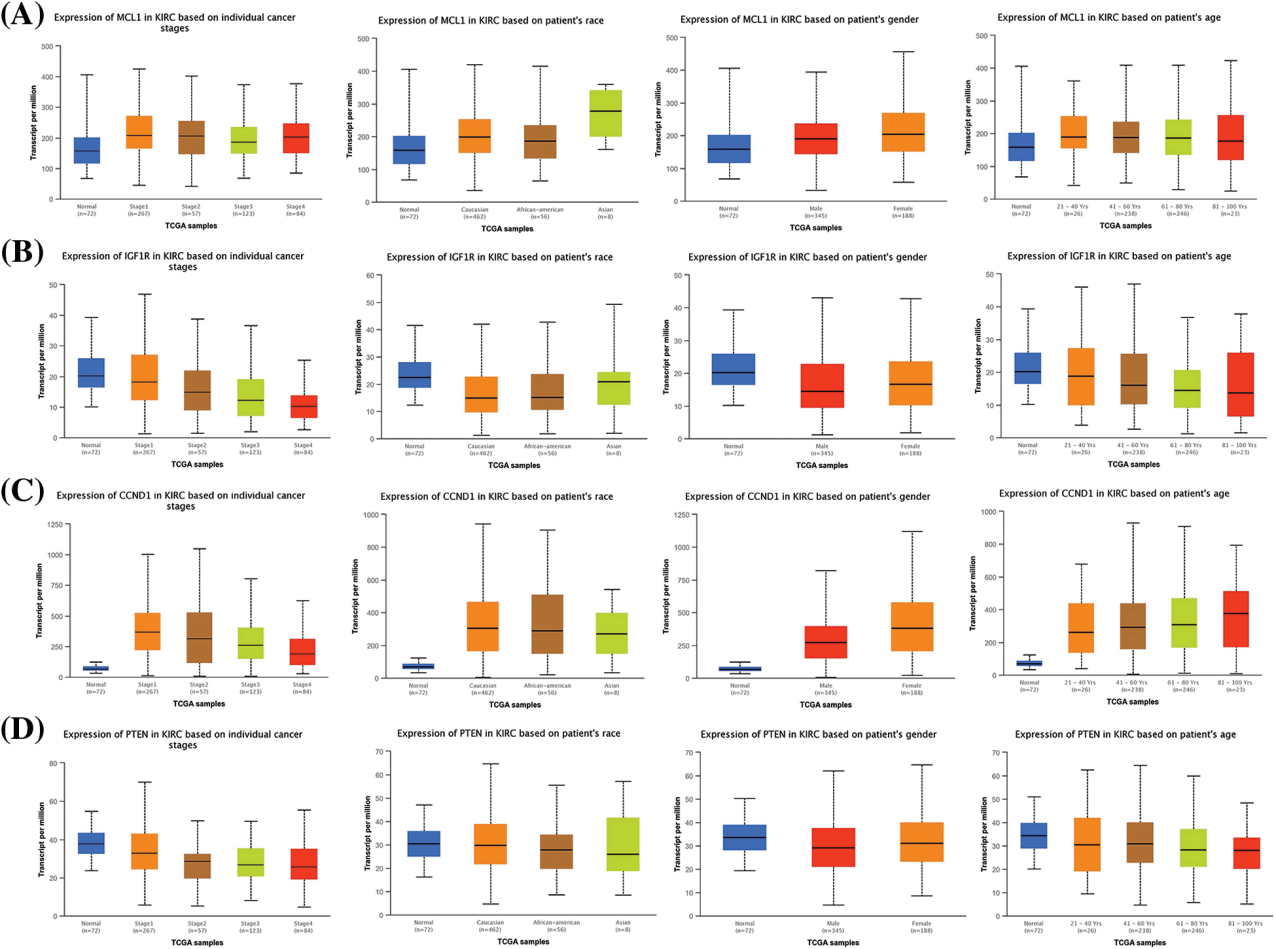
Expression profiling of MCL1, IGF1R, CCND1, and PTEN in KIRC samples of different clinical variables relative to controls via UALCAN. (A) Expression profiling of MCL1 in KIRC samples of different clinical variables, (B) expression profiling of IGF1R in KIRC samples of different clinical variables, (C) expression profiling of CCND1 in KIRC samples of different clinical variables, and (D) expression profiling of PTEN in KIRC samples of different clinical variables.

Furthermore, we also investigated the potential implications of dysregulation of these genes in KIRC patients with different clinicopathological parameters. Information on cancer stage, race, gender, and age was retrieved from UALCAN, and these parameters were analyzed in relation to mRNA expression of the hub genes in the KIRC cohort. Results revealed a significant up-regulation of MCL1 and CCND1, and a significant down-regulation of IGF1R and PTEN in KIRC patients with varying cancer stages, races, genders, and age groups compared to normal controls (Fig. 3).

### Expression verification and survival analyses of MCL1, IGF1R, CCND1, and PTEN

**Figure 4:**
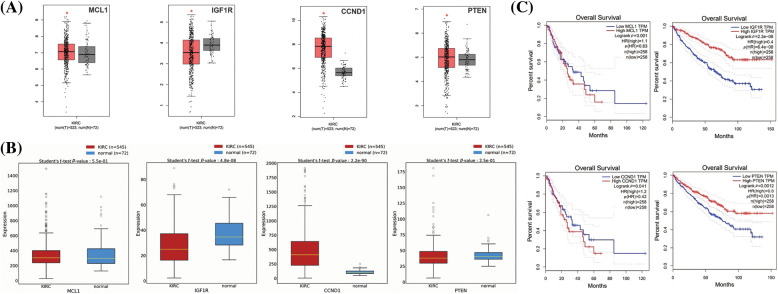
Expression validation and survival analysis of MCL1, IGF1R, CCND1, and PTEN. (A) Expression validation of MCL1, IGF1R, CCND1, and PTEN in KIRC and normal samples via GEPIA database, *p < 0.05. (B) Expression validation of MCL1, IGF1R, CCND1, and PTEN in KIRC and normal samples via OncoDB database, and (C) survival analysis of MCL1, IGF1R, CCND1, and PTEN in KIRC and normal samples via GEPIA database.

In order to enhance the validity of our findings, we incorporated two additional databases, namely GEPIA and OncoDB, to perform expression verification analysis. For this purpose, we evaluated the expression levels of the MCL1, IGF1R, CCND1, and PTEN, as well as their impact on survival in KIRC and control tissues (Fig. 4). Our results indicated that MCL1 and CCND1 exhibited significantly higher mRNA expression levels (*p* < 0.05) while IGF1R and PTEN mRNA levels were significantly lower (*p* < 0.05) in KIRC samples compared to healthy samples (Figs. 4A and 4B). Furthermore, we employed the GEPIA database to investigate the hub genes’ impact on overall survival (OS), and the analysis revealed a significant (*p* < 0.05) correlation between dysregulated MCL1, IGF1R, CCND1, PTEN, and poor OS for KIRC patients (Fig. 4C).

### Subcellular localization and protein expression validation analyses of MCL1, IGF1R, CCND1, and PTEN

**Figure 5:**
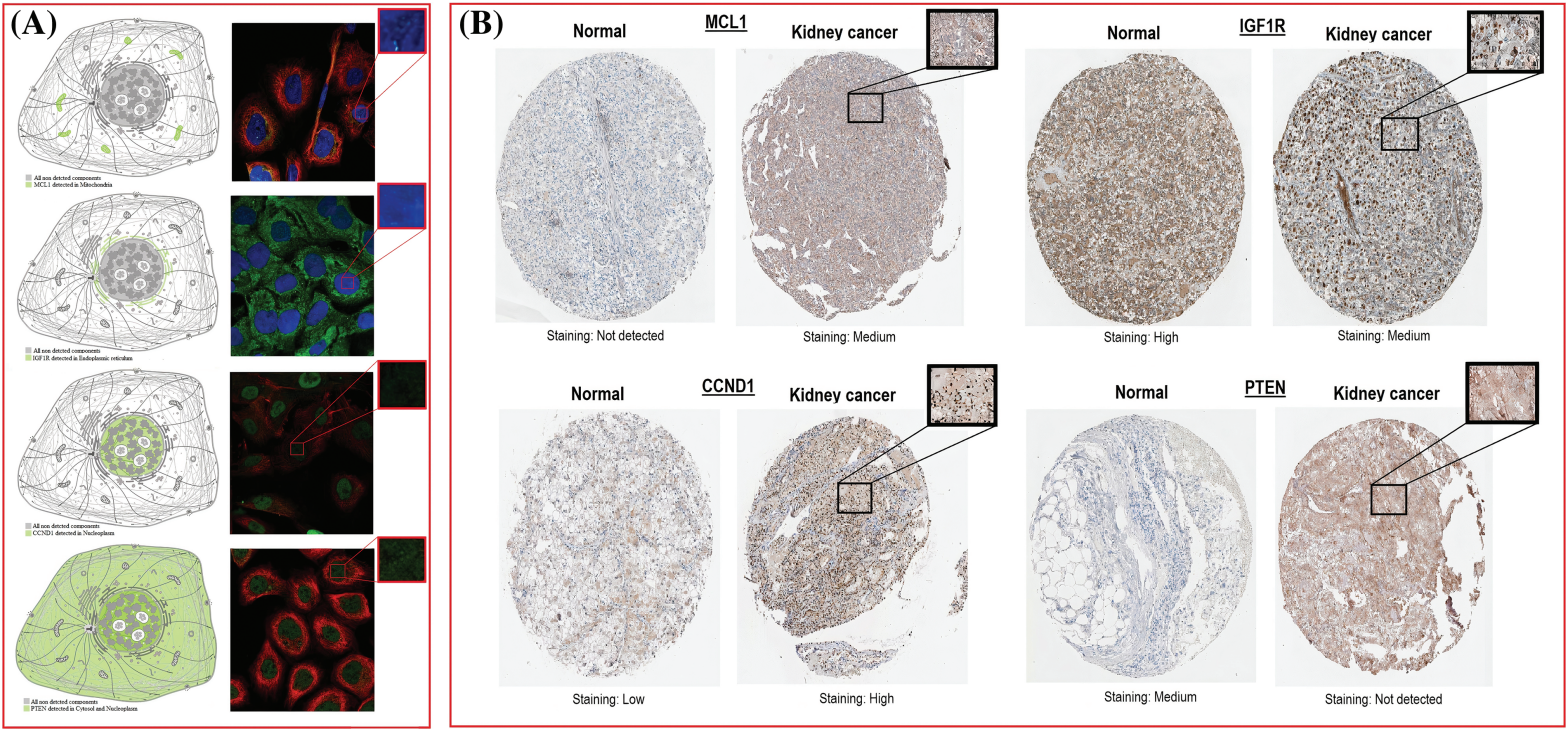
Subcellular localization and protein expression validation of MCL1, IGF1R, CCND1, and PTEN via HPA database. (A) Subcellular localization prediction of MCL1, IGF1R, CCND1, and PTEN, and (B) protein expression analysis of MCL1, IGF1R, CCND1, and PTEN in KIRC and normal samples.

The location of MCL1, IGF1R, CCND1, and PTEN within KIRC cells was determined using HPA. MCL1 was found to be primarily located in mitochondria (Fig. 5A), IGF1R was localized in the endoplasmic reticulum (Fig. 5A), CCND1 was enriched in the nucleoplasm (Fig. 5A), and PTEN was present in both the cytosol and nucleoplasm (Fig. 5A). The expressions of these proteins were confirmed in KIRC samples through immunohistochemistry (IHC) analysis using data obtained from HPA. The IHC results revealed that MCL1 and CCND1 had higher expressions (staining = medium and high) in KIRC samples (Fig. 5B) compared to control samples (staining = not detected and low) (Fig. 5B). Conversely, the expressions of IGF1R and PTEN were lower (staining = medium and not detected) in KIRC samples (Fig. 5B) compared to control samples (staining = high and medium) (Fig. 5B).

### Effect of DNA mutations and promoter methylation on MCL1, IGF1R, CCND1, and PTEN dysregulation and KIRC patients’ survival

**Figure 6:**
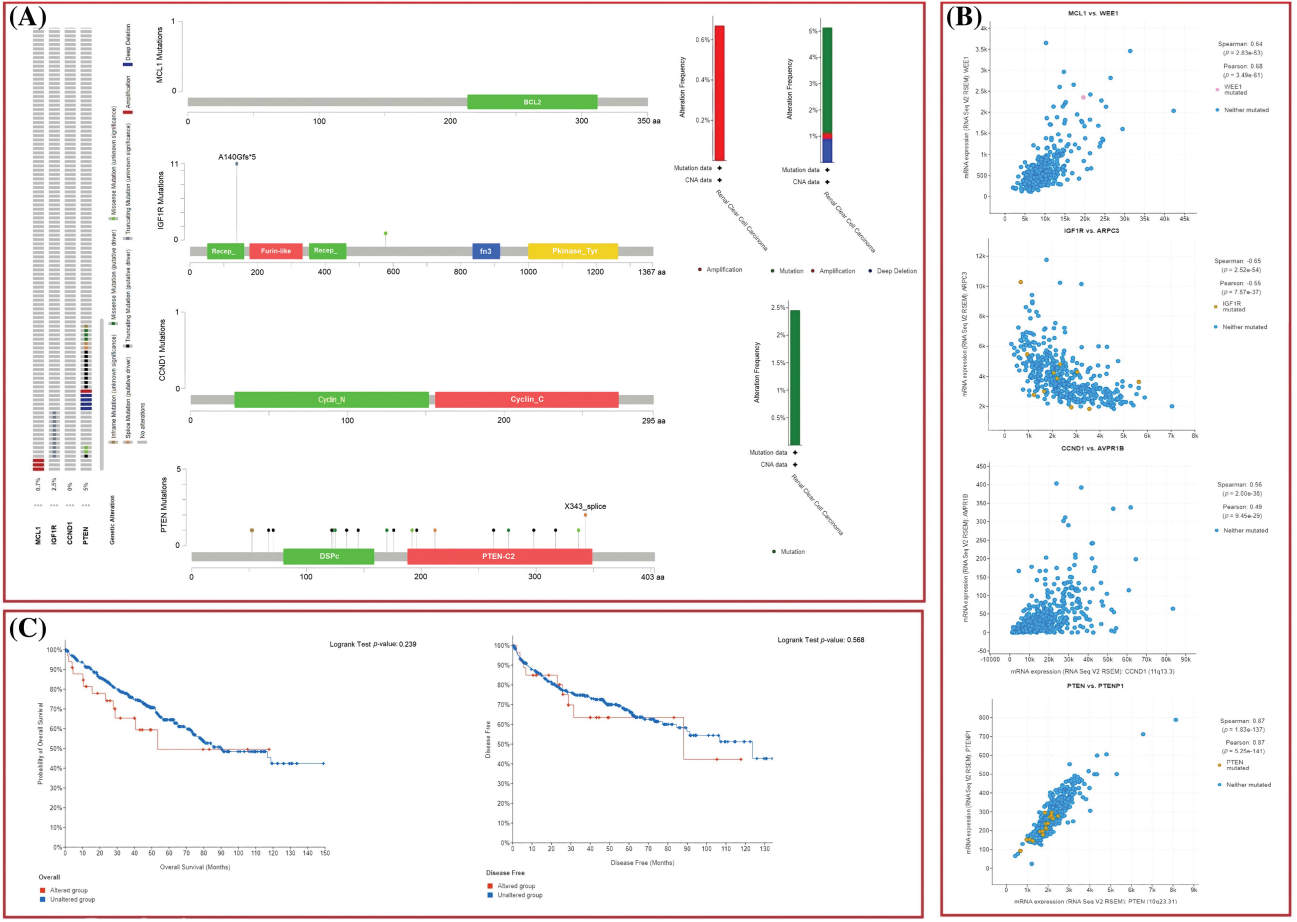
Exploration of genetic alteration frequencies, mutational hotspots, OS, DFS, and co-expressed gene analyses of MCL1, IGF1R, CCND1, and PTEN in KIRC samples via cBioPortal. (A) Types, frequencies, and location of the genetic alterations in MCL1, IGF1R, CCND1, and PTEN, (B) identification of co-expressed genes with TYROBP, PTPRC, LCP2, and ITGB2 in KIRC samples, and (C) OS and DFS analysis of MCL1, IGF1R, CCND1, and PTEN in genetically altered and unaltered KIRC groups.

**Figure 7:**
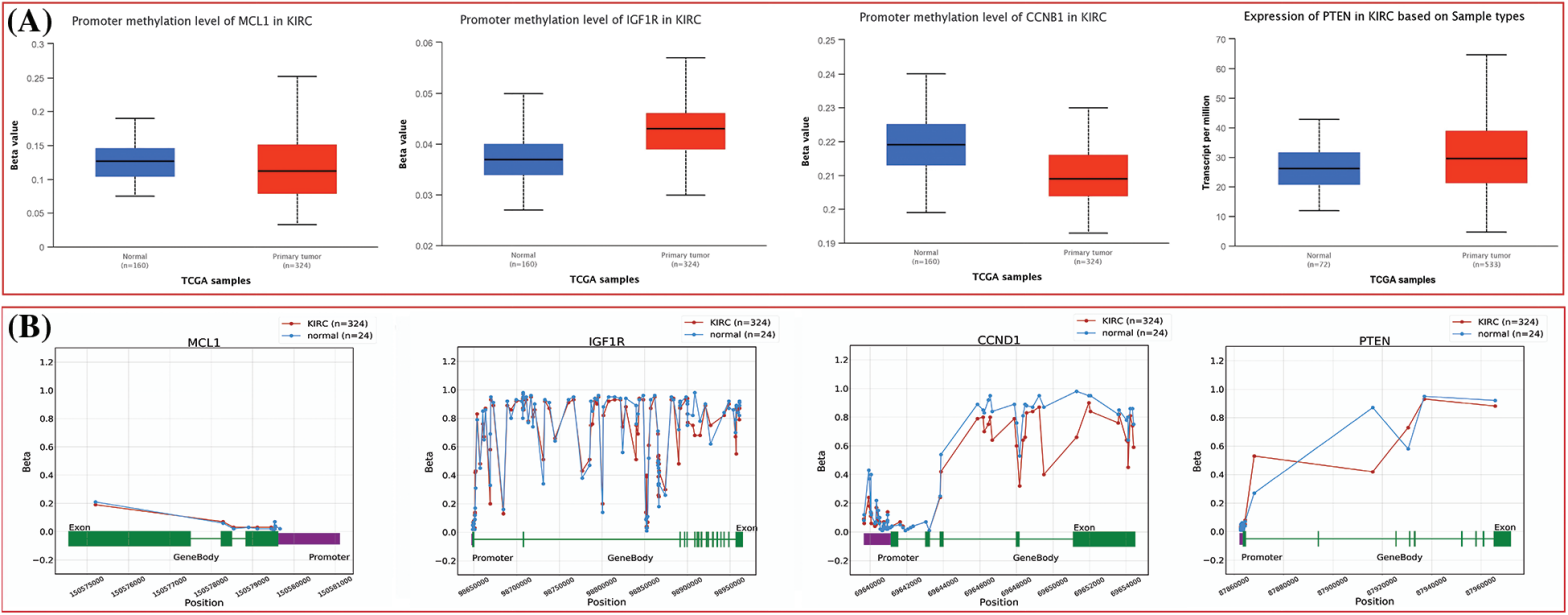
Methylation status exploration of MCL1, IGF1R, CCND1, and PTEN via UALCAN and OncoDB in KIRC and normal samples. (A) Methylation status exploration of MCL1, IGF1R, CCND1, and PTEN via UALCAN, and (B) methylation status exploration of MCL1, IGF1R, CCND1, and PTEN via OncoDB.

The dysregulation of MCL1, IGF1R, CCND1, and PTEN expressions was found to be correlated with various clinical parameters of KIRC and the worst OS. Therefore, exploring the potential regulatory mechanisms involved in the overexpression of these hub genes could have clinical significance. Firstly, we used the cBioPortal database to identify genetic mutations in the hub genes in the TCGA KIRC cohort. Low genetic alteration frequencies were detected in MCL1 (0.7%), IGF1R (0%), and CCND1 (2.5%), indicating their lesser involvement in hub gene expression regulation (Fig. 6A). PTEN had a higher genetic alteration frequency of 5%, which was linked to its down-regulation in KIRC patients (Fig. 6A). The top co-expressed genes were identified through cBioPortal, which included MCL1-WEE1, IGF1R-ARPC3, CCND1-AVPRB1, and PTEN-PTENP1 (Fig. 6B). Genetic alterations in the hub genes were also associated with the worst OS of KIRC patients (Fig. 6C). Finally, promoter methylation levels of the hub genes in normal control and KIRC samples were examined using the UALCAN and OncoDB databases. We found that the promoter methylation levels of MCL1 and CCNB1 were lower, while the promoter methylation levels of IGF1R and PTEN were higher in KIRC samples than in normal tissues (Fig. 7). Thus, abnormal promoter DNA methylation levels may lead to aberrant expression of MCL1, IGF1R, CCND1, and PTEN in KIRC.

### GO and biological pathways analysis

**Figure 8:**
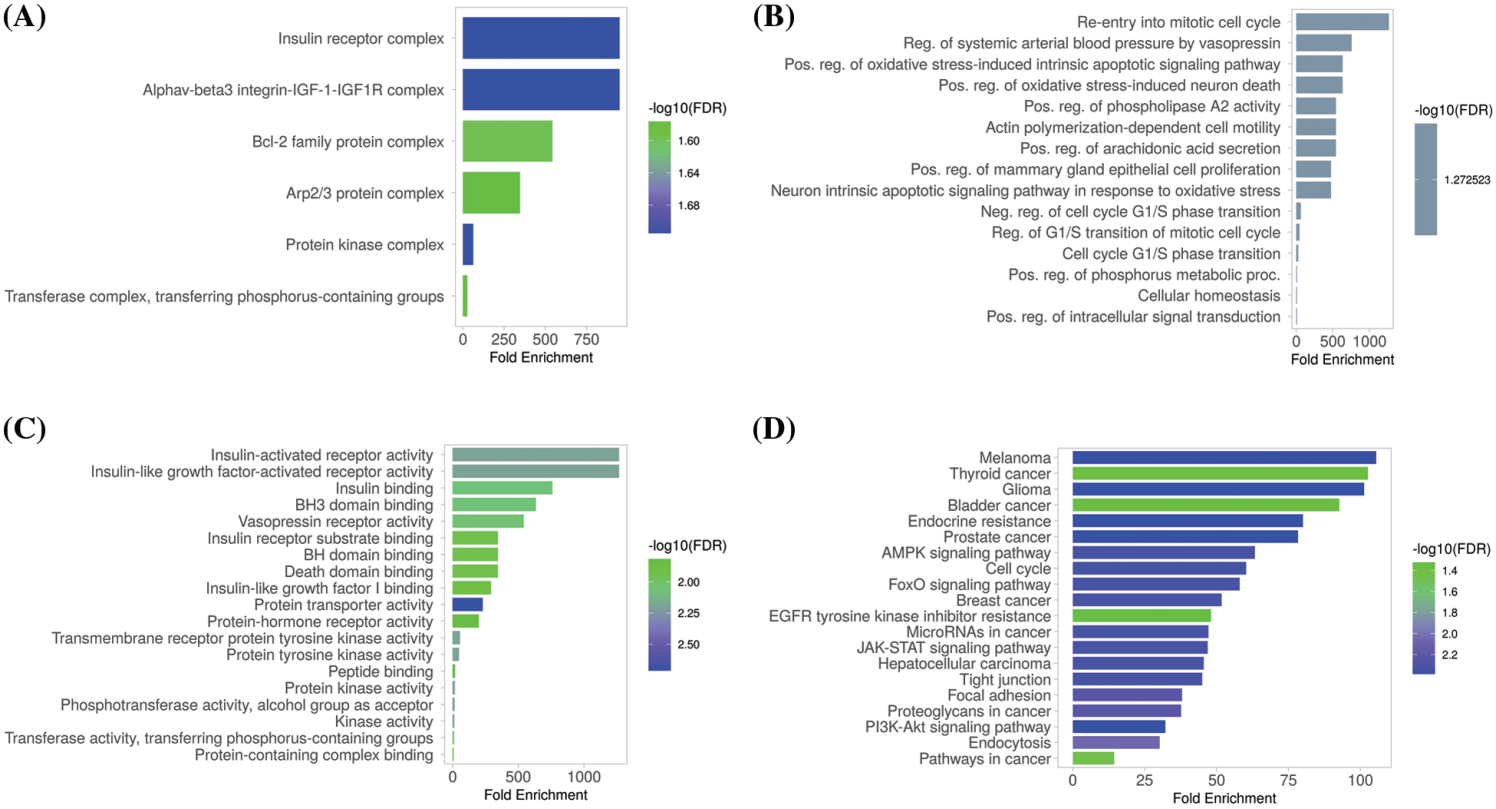
Gene enrichment analysis of MCL1, IGF1R, CCND1, and PTEN. (A) MCL1, IGF1R, CCND1, and PTEN associated CC terms, (B) MCL1, IGF1R, CCND1, and PTEN associated BP terms, (C) MCL1, IGF1R, CCND1, and PTEN associated MF terms, and (D) MCL1, IGF1R, CCND1, and PTEN associated KEGG terms.

The GSEA program was used to conduct GO and KEGG analyses of the identified hub genes. We obtained GO functional enrichments of the MCL1, IGF1R, CCND1, and PTEN genes with a *p*-value < 0.05. GO and KEGG pathways in KIRC. In the CC, “Insulin receptor complex, Alphav-beta integrin-IGF-1-IGF1R complex, Bcl-2 family protein complex, and Arp2/3 protein complex”, etc., were significantly associated with the analyzed genes (Fig. 8A). Concerning MF, the “Re-entry into mitotic cell cycle, Reg. of systemic arterial blood pressure by vasopressin, and Pos, Reg. phospholipase A2 activity”, etc., were closely associated the hub genes (Fig. 8B). In BP, some vital functions including “Insulin-activated receptor activity, Insulin-like growth factor-activated receptor activity, Insulin Binding, and BH3 binding”, etc., were significantly associated with hub genes (Fig. 8C). Hub genes and their co-expressed genes associated KEGG pathways include “Melanoma, Thyroid cancer, Glioma, Bladder cancer, and Prostate cancer”, etc. (Fig. 8D).

### Immune cell analysis of MCL1, IGF1R, CCND1, and PTEN

**Figure 9:**
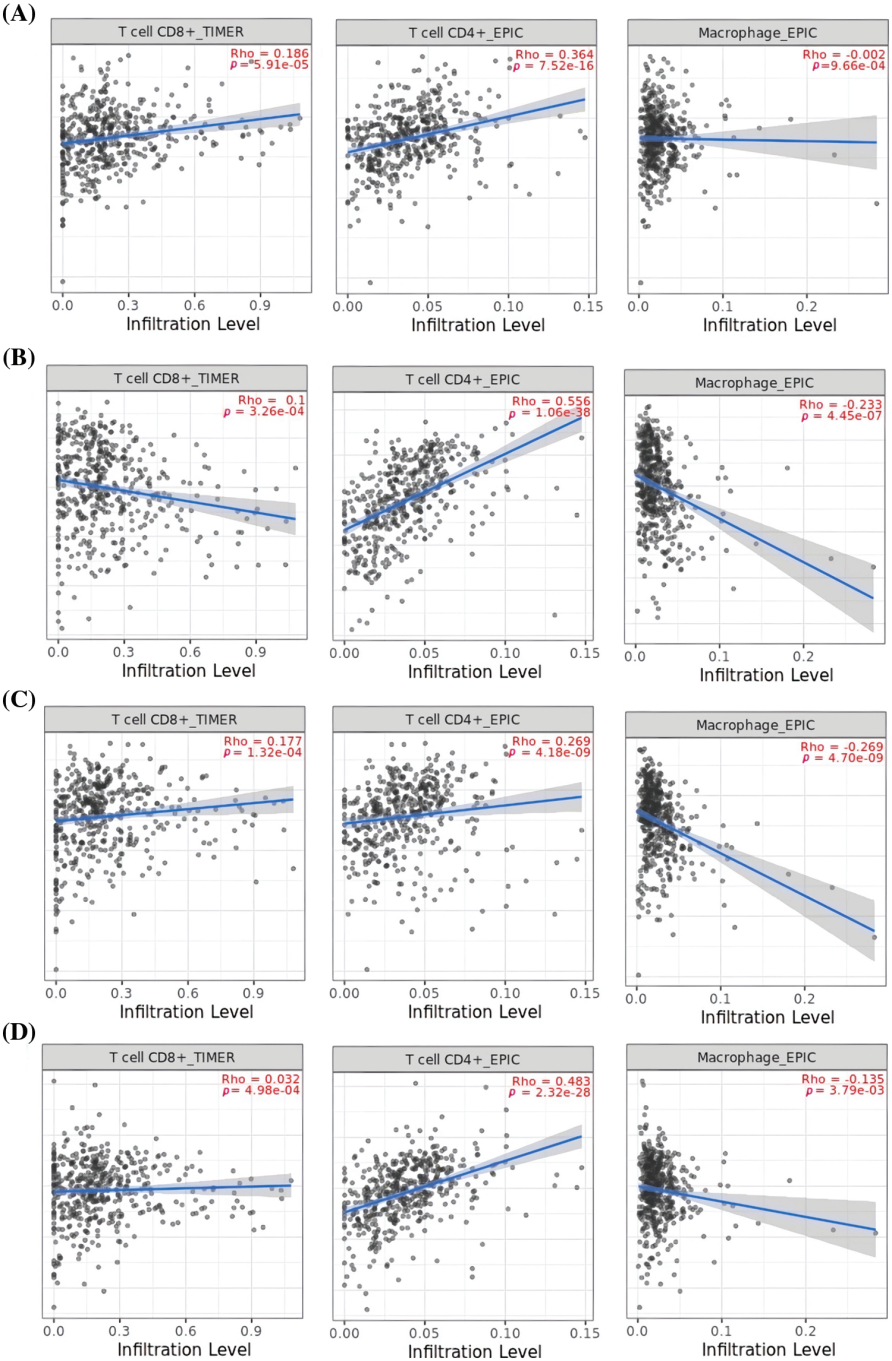
Correlation analysis of MCL1, IGF1R, CCND1, and PTEN hub genes expression with different immune cells (CD8+ T, CD4+ T, and Macrophages) infiltration level. (A) MCL1, (B) IGF1R, (C) CCND1, and (D) PTEN.

Next, TIMER was utilized to uncover associations between the expression of MCL1, IGF1R, CCND1, and PTEN genes, and immune cell influx (CD8+ T, CD4+ T, and macrophages). A significant positive correlation (*p* < 0.05) was observed between CD8+ T and CD4+ T cell infiltration and the expression levels of MCL1, IGF1R, CCND1, and PTEN genes (Fig. 9), while a negative correlation was observed with macrophages (Fig. 9).

### miRNA network of the MCL1, IGF1R, CCND1, and PTEN

**Figure 10:**
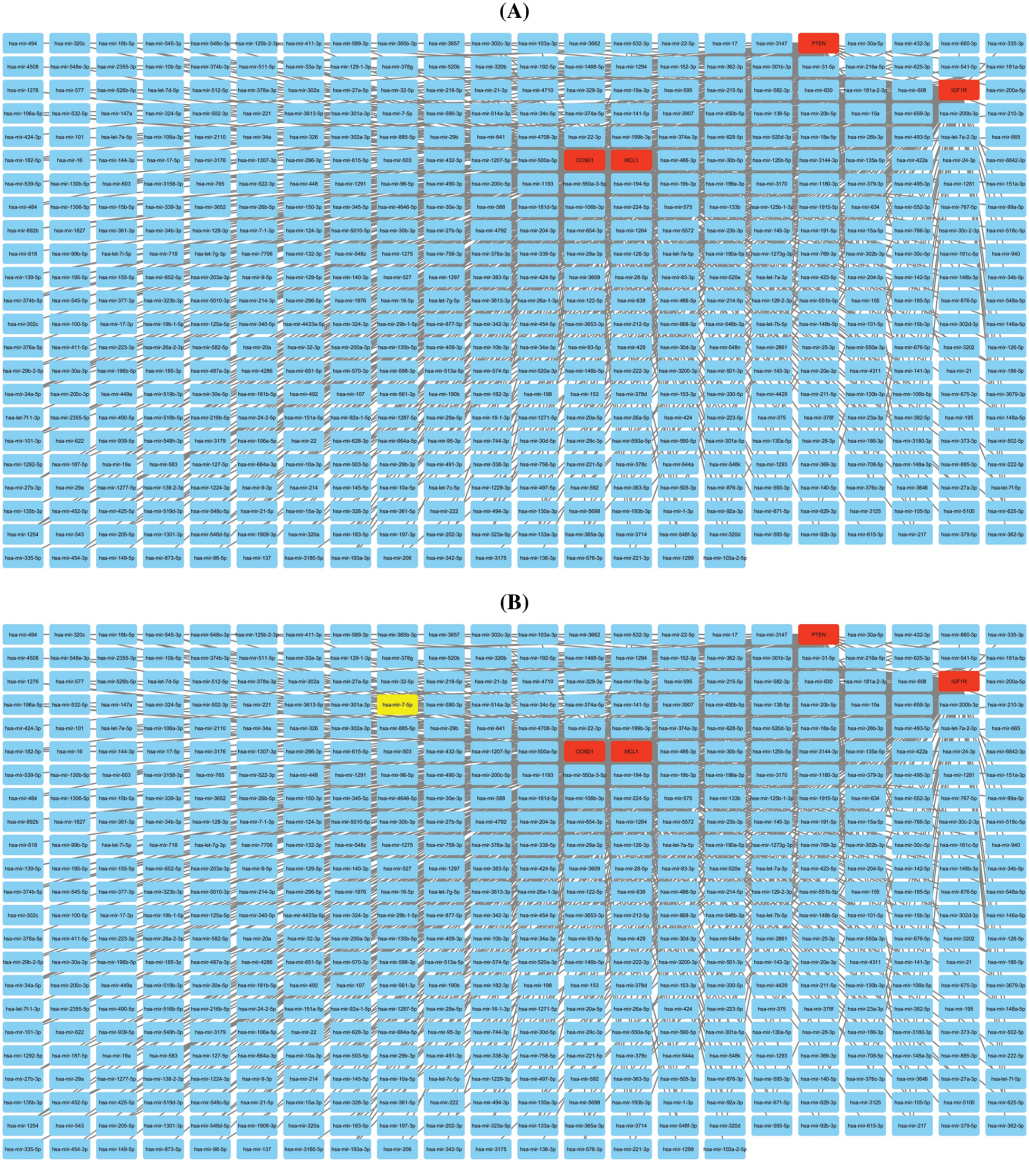
miRNA-mRNA co-regulatory network of MCL1, IGF1R, CCND1, and PTEN hub genes. (A) A PPI of miRNAs targeting hub genes, and (B) a PPI highlighting most important miRNA (hsa-mir-17-5p) targeting all hub genes. Light blue color nodes: miRNAs, red color nodes: mRNAs, and green color node: hsa-mir-17-5p.

Additionally, we established a miRNA-mRNA co-regulatory network of the aforementioned genes through ENCORI and Cytoscape. The constructed miRNA-mRNA co-regulatory network had a total of 454 miRNAs and 4 mRNAs. Based on the degree method, Cytohubba analysis further identified a miRNA (has-mir-17-5p) as a potential inducer of KIRC, as it targets all hub genes simultaneously. These findings suggest that the identified axis of has-mir-27a-3p and MCL1, IGF1R, CCND1, and PTEN genes may have a role in drug resistance development across KIRC (Fig. 10).

### Drug prediction analysis of the MCL1, IGF1R, CCND1, and PTEN

For KIRC patients suffering from cisplatin drug resistance, the use of alternative drugs for medical treatment is the first option. Therefore, a selection of appropriate alternate candidate drugs is required. In the current study, via the DrugBank database, we explored some potential drugs, that can reverse the gene expressions of MCL1, IGF1R, CCND1, and PTEN hub genes. Alvocidib, Estradiol, Tretinoin, Capsaicin, Dronabinol, Metribolone, Calcitriol, Acetaminophen, Acitretin, Cyclosporine, Azacitidine, Genistein, and Resveratrol drugs (Table 1) could be useful to target MCL1, IGF1R, CCND1, and PTEN hub genes to counteract cisplatin resistance once clinical significance is established via preclinical/clinical studies.

**Table 1:** DrugBank-based MCL1, IGF1R, CCND1, and PTEN associated drugs

Sr. No.	Hub gene	Drug name	Effect	Reference	Group
1	MCL1	Alvocidib	Decrease expression of MCL1 mRNA	A20630	Approved
		Estradiol		A21152	
		Tretinoin		A20405	
2	IGF1R	Capsaicin	Increase expression of IGF1R mRNA	A21513	Approved
		Dronabinol		A22085	
		Metribolone		A21310	
		Calcitriol		A22301	
3	CCND1	Acetaminophen	Decrease expression of CCND1 mRNA	A20420	Approved
		Acitretin		A20453	
		Cyclosporine		A20661	
4	PTEN	Estradiol	Increase expression of PTEN mRNA	A21179	Approved
		Azacitidine		A20985	
		Genistein		A22790	
		Resveratrol		A22790	
		Tretinoin		A24453	

### In vitro validation of cisplatin resistance-inducing genes via targeted bisulfide-seq

**Figure 11:**
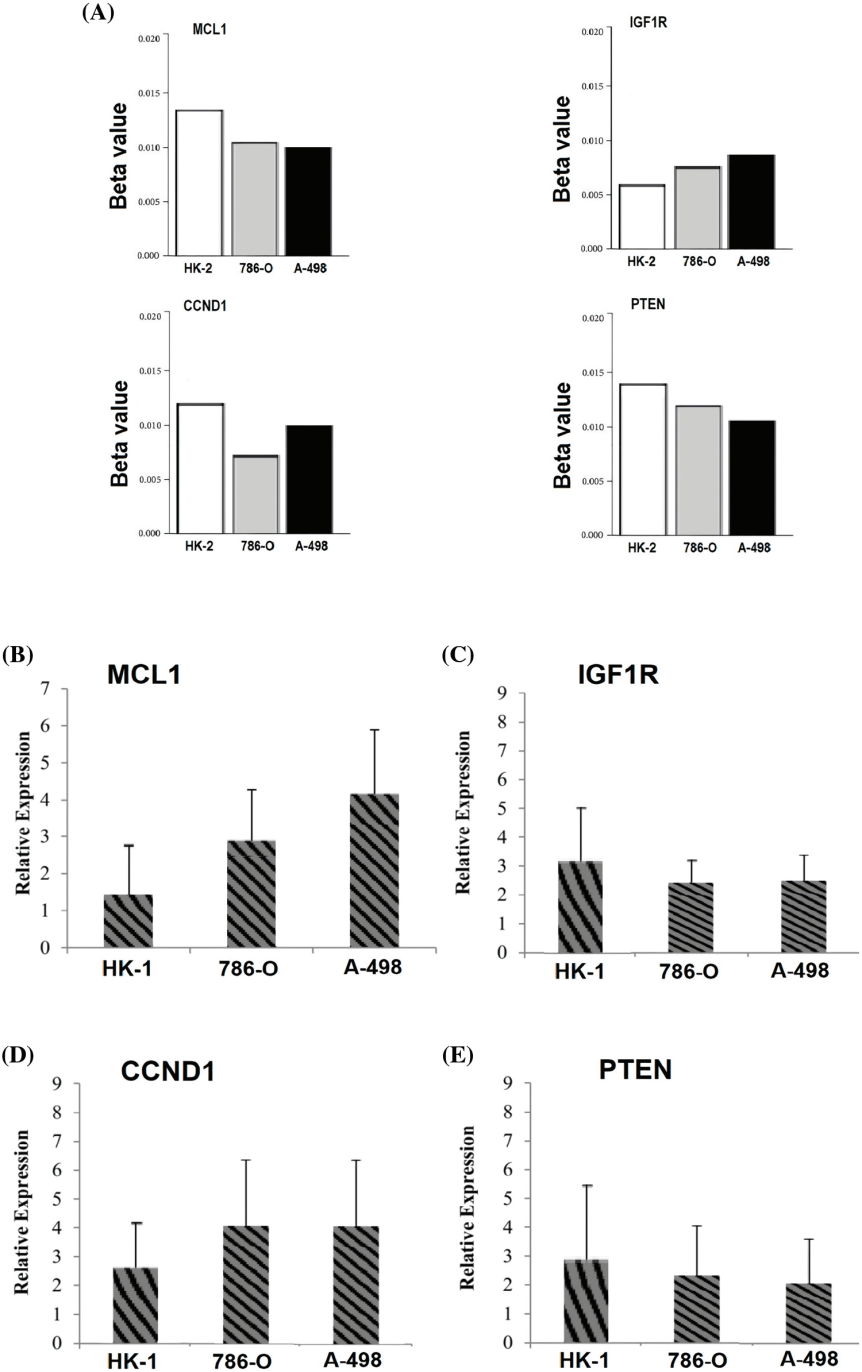
Validating MCL1, IGF1R, CCND1, and PTEN promoter methylation and expression levels using cisplatin resistant cell lines (786-O and A-498), and the normal renal tubular epithelial cell line (HK-2) through targeted bisulfite-seq and RT-qPCR analyses. (A) Beta value based promoter methylation level of hub genes, (B), relative expression of MCL1, (C) relative expression of IGF1R, (D) relative expression of CCND1, and (E) relative expression of PTEN. The x-axis represents different groups, and the y-axis represents relative promoter methylation and expression of genes.

In the current study, by performing targeted bisulfite-seq analyses of 2 cisplatin-resistant RCC cell lines, including 786-O and A-498, and the normal renal tubular epithelial cell line HK-2, methylation levels of the MCL1, IGF1R, CCND1, and PTEN were validated. Methylation levels of these genes were validated using beta values. As shown in Fig. 11A, it was noticed that beta values of MCL1 and CCND1 were lower, while beta values of IGF1R and PTEN were higher in the cisplatin-resistant RCC cell lines (786-O and A-498) as compared to the normal control cell line (HK-2).

### RT-qPCR validation analysis of MCL1, IGF1R, CCND1, and PTEN

To further validate the aforementioned bioinformatics analysis, the mRNA expression levels of these hub genes (MCL1, IGF1R, CCND1, and PTEN) were obtained by RT-qPCR experiment in cisplatin resistant RCC cell lines (786-O and A-498) and a normal control cell line (HK-2). As suggested in Fig. 11B, there were notable differences between the cisplatin resistant 786-O and A-498 cell lines and the HK-2 cell line in all four hub genes, including MCL1, IGF1R, CCND1, and PTEN (Fig. 11B). Interestingly, MCL1 and CCND1 genes had up-regulation, while IGF1R and PTEN had notable down-regulation in the cisplatin-resistant RCC cell line relative to the normal control cell line (HK-2) (Fig. 11B) as previously predicted by the TCGA dataset analysis.

## Discussion

Cisplatin resistance refers to the ability of cancer cells to become resistant to the effects of cisplatin, to treat a variety of cancers, including KIRC [52]. Cisplatin resistance can develop due to a variety of mechanisms, including dysregulation of important genes, and changes in cellular signaling pathways [53]. According to scientific research, proteins with homologous similarities from phylogenetic evolution tend to conserve their protein domains, making them essentially alike [54]. This means that domain-conserved proteins may interact with each other when influenced by external factors such as xenobiotics [55]. In drug resistance scenarios, targeting a single protein may activate or inhibit the function of homologous proteins to compensate for the loss of the targeted protein’s function [56]. However, there is a need for more clarification and declaration of the protein-protein interactions among the trigger genes linked to cisplatin resistance in KIRC. To this end, the current study sets out to investigate the protein-protein interaction among the genes and identify some important genes linked to cisplatin resistance in KIRC. The identified hub genes will help in understanding the molecular mechanisms underlying cisplatin resistance and developing new therapeutic strategies for KIRC patients. Therefore, this study was initiated for identifying cisplatin resistance-associated biomarker hub genes across KIRC through *in silico* and *in vitro* methodologies.

To do so, initially, 124 cisplatin resistance-linked genes were identified from the published literature. Then, PPI of the identified genes was constructed and screened out via Cytoscape software to identify hub genes. Based on the degree method, the MCL1, IGF1R, CCND1, and PTEN genes were identified as the most prominent drug resistance-associated genes in KIRC.

The MCL1 gene encodes for a protein called myeloid cell leukemia 1, which plays a crucial role in regulating programmed cell death, or apoptosis [57,58]. In normal cells, MCL1 helps maintain a balance between cell growth and cell death [59]. However, in cancer cells, MCL1 expression is often up-regulated, leading to increased cell survival and resistance to chemotherapy drugs. This makes MCL1 an attractive target for cancer therapy development [60]. Several studies have shown that targeting MCL1 can induce apoptosis in cancer cells and sensitize them to chemotherapy [61,62]. Moreover, MCL1 has been implicated in resistance to a range of cancer therapies, including those targeting the BCL-2 family of proteins, which are also involved in apoptosis regulation [63,64]. Therefore, there is significant interest in developing drugs that can inhibit MCL1 expression or function as a potential cancer treatment strategy. In summary, the MCL1 gene plays a critical role in cancer by promoting cell survival and resistance to chemotherapy. Targeting MCL1 represents a promising therapeutic approach for overcoming resistance to cancer treatment and improving patient outcomes.

The IGF1R (Insulin-like Growth Factor 1 Receptor) gene encodes for a transmembrane receptor protein that binds to insulin-like growth factors (IGFs) and plays a key role in cell growth and survival [65,66]. In normal cells, the IGF1R signaling pathway is tightly regulated, but in cancer cells, it can become dysregulated, leading to uncontrolled cell growth and proliferation [67]. The overexpression of IGF1R has been observed in a variety of cancers, including breast, lung, prostate, and colon cancer [68], making it an attractive target for cancer therapy. Studies have shown that blocking the IGF1R pathway can inhibit cancer cell growth and promote apoptosis [69,70]. In preclinical studies, IGF1R inhibitors have demonstrated promising anti-tumor activity, particularly in combination with other cancer therapies [68]. Additionally, the IGF1R pathway has been linked to resistance to several cancer treatments, including chemotherapy and targeted therapies, highlighting its potential as a therapeutic target for overcoming drug resistance [68]. Despite initial enthusiasm, clinical trials of IGF1R inhibitors have shown mixed results, with some trials showing limited efficacy and others showing promising results in specific patient populations [71]. Overall, the IGF1R gene is an important player in cancer pathogenesis and a promising target for cancer therapy development.

The CCND1 gene, also known as cyclin D1, plays a crucial role in the cell cycle by regulating the G1/S transition [72,73]. However, overexpression of CCND1 has been linked to drug resistance in cancer cells [74]. Studies have shown that increased expression of CCND1 in cancer cells can lead to the activation of various drug-resistance pathways, such as the up-regulation of drug transporters and anti-apoptotic proteins [75]. Furthermore, inhibition of CCND1 has been found to sensitize cancer cells to chemotherapy and radiation therapy [76]. Therefore, targeting the CCND1 gene may offer a promising strategy to overcome drug resistance in cancer treatment. Overall, the CCND1 gene appears to be a key player in drug resistance in cancer cells, representing a valuable target for the development of novel cancer treatments.

The PTEN gene is a tumor suppressor gene that plays a critical role in regulating cell growth and proliferation [77]. PTEN is often mutated or deleted in many different cancers, leading to the loss of its function and uncontrolled cell growth [78]. Recent studies have also shown that the loss of PTEN function is associated with drug resistance in cancer cells [79,80]. In particular, PTEN loss has been linked to resistance to targeted therapies such as tyrosine kinase inhibitors and immune checkpoint inhibitors [81]. The mechanisms by which PTEN loss confers drug resistance are not yet fully understood but may involve altered signaling pathways, changes in the tumor microenvironment, and alterations in DNA repair mechanisms. Understanding the role of PTEN in drug resistance may lead to the development of novel therapeutic strategies to overcome this resistance in cancer patients.

We next analyzed and validated the mRNA and protein expression levels of MCL1, IGF1R, CCND1, and PTEN using UALCAN, GEPIA, OncoDB, and HPA databases on TCGA datasets. Our research took into account different factors such as cancer stages, races, genders, ages, and pathological data. Analysis of the UALCAN, GEPIA, and OncoDB databases revealed a significant up-regulation of MCL1 and CCND1 mRNA expressions, and a significant down-regulation of IGF1R and PTEN mRNA expressions in KIRC samples compared to controls. We also found that the protein expression of MCL1, IGF1R, CCND1, and PTEN in KIRC tissue samples was consistent with their corresponding mRNA expression levels using the HPA database and immunohistochemical staining. Therefore, we infer that the high expressions of MCL1 and CCND1 and low expressions of IGF1R and PTEN may play critical roles in developing cisplatin drug resistance across KIRC. The analysis of promoter methylation has revealed that an abnormal pattern of methylation significantly contributes to the dysregulation of MCL1, IGF1R, CCND1, and PTEN expression in KIRC patients compared to normal controls. MCL1 methylation has been associated with poor overall survival in KIRC patients [82], while IGF1R and CCND1 methylation have been linked to tumor aggressiveness [75,83]. PTEN methylation, on the other hand, has been associated with the loss of tumor suppressor function in KIRC [84]. Subsequently, we utilized the Kaplan–Meier plotter and discovered that a high expression of MCL1 and CCND1, along with low expressions of IGF1R and PTEN, could lead to a shorter survival time for KIRC patients. Therefore, the expression levels of MCL1, IGF1R, CCND1, and PTEN can serve as an independent risk factor for a poor prognosis of KIRC.

The TIMER analysis revealed a significant correlation between the expression of MCL1, IGF1R, CCND1, and PTEN and the presence of CD8+ T cells, CD4+ T cells, and macrophages. The CD8+ T and CD4+ T cells are the most abundant types of T lymphocytes in tumor microenvironments and capable of eliminating tumor cells, their differentiation into dysfunctional states may render them ineffective at responding to immunotherapy [85,86]. This suggests that the dysregulation of MCL1, IGF1R, CCND1, and PTEN expression could be responsible for KIRC aggressiveness and resistance to immunotherapy by impairing the functionality of CD8+ T and CD4+ T cells. Furthermore, the miRNA hsa-mir-17-5p was found to be a common modulator of MCL1, IGF1R, CCND1, and PTEN hub gene expression in KIRC patients through miRNA network analysis. The cisplatin resistance-associated role of hsa-mir-17-5p is well-established in various types of cancer, including colorectal cancer, head and neck cancer, and leukemia [87,88]. However, this study is the first to report the cisplatin resistance-associated role of hsa-mir-17-5p as a modulator of MCL1, IGF1R, CCND1, and PTEN hub genes. Lastly, KEGG analysis further validated the connection of MCL1, IGF1R, CCND1, and PTEN hub genes with different cisplatin resistance-associated pathways, such as Endocrine resistance, AMPK signaling pathway, and the Foxo signaling pathway [89–91].

## Conclusion

This study revealed that MCL1, IGF1R, CCND1, and PTEN, are the central genes implicated in cisplatin resistance in KIRC. These genes demonstrated significant expression variability and had an impact on the OS of KIRC patients. These genes were also found modulating multiple cisplatin-associated pathways. Targeting MCL1, IGF1R, CCND1, and PTEN genes with different other drugs explored via the present study, such as palbociclib, methotrexate, bortezomib, fluorouracil, sorafenib, dasatinib, carboplatin, paclitaxel, gemcitabine, imatinib, doxorubicin, and vorinostat, may have the potential to overcome cisplatin drug resistance in KIRC.

## Data Availability

No datasets were generated or analyzed during the current study.
